# Newcastle disease virus employs macropinocytosis and Rab5a-dependent intracellular trafficking to infect DF-1 cells

**DOI:** 10.18632/oncotarget.13345

**Published:** 2016-11-15

**Authors:** Lei Tan, Yuqiang Zhang, Yuan Zhan, Yanmei Yuan, Yingjie Sun, Xusheng Qiu, Chunchun Meng, Cuiping Song, Ying Liao, Chan Ding

**Affiliations:** ^1^ Department of Avian Diseases, Shanghai Veterinary Research Institute, Chinese Academy of Agricultural Sciences, Shanghai 200241, P.R. China; ^2^ Jiangsu Co-innovation Center for Prevention and Control of Important Animal Infectious Diseases and Zoonoses, Yangzhou, 225009, P.R. China

**Keywords:** newcastle disease virus, endocytosis, clathrin, macropinocytosis, Rab5a

## Abstract

Oncolytic Newcastle disease virus (NDV) reportedly employs direct fusion of the viral envelope with the plasma membrane and caveolae-dependent endocytosis to enter cells. Here, we show that macropinocytosis and clathrin-mediated endocytosis are involved in NDV entry into a galline embryonic fibroblast cell line. Upon specific inhibition of clathrin assembly, GTPase dynamin, Na^+^/H^+^ exchangers, Ras-related C3 botulinum toxin substrate 1, p21 activated kinase 1 or protein kinase C, entry of NDV and its propagation were suppressed. NDV entry into cells triggers Rac1-Pak1 signaling and elicits actin rearrangement and plasma membrane ruffling. Moreover, NDV internalization within macropinosomes and trafficking involve Rab5a-positive vesicles. This is the first report demonstrating that NDV utilizes clathrin-mediated endocytosis and macropinocytosis as alternative endocytic pathways to enter cells. These findings shed new light on the molecular mechanisms underlying NDV entry into cells, and provide potential targets for NDV-mediated therapy in cancer.

## INTRODUCTION

Newcastle disease virus (NDV) is an avian paramyxovirus. Its virions are pleomorphic, but mostly spherical with a diameter of approximately 100 nm [1]. NDV causes respiratory disease in domestic fowl that can lead to huge economic losses in the poultry industry [2]. It is thought that NDV enters cells after direct fusion with the plasma membrane through a pH-independent mechanism. In addition, NDV may enter host cells through a caveolae-mediated endocytosis (CavME) pathway [3], as well as through other endocytotic routes that are not yet well understood.

The mainly endocytic pathways include clathrin-mediated endocytosis (CME), CavME, macropinocytosis and phagocytosis, among others [4]. With CME, clathrin is assembled on the cytoplasmic face of the plasma membrane to form a clathrin-coated pit (CCP). Internalization of the virus-receptor complexes occurs within CCPs, which invaginate toward the cytoplasmic side and dissociate to form clathrin-coated vesicles containing the endocytic cargo, which are then delivered into endosomes. Semliki forest virus [5], vesicular stomatitis virus [6, 7], egg drop syndrome virus [8], and human immunodeficiency virus-1 (HIV-1) [9] all use the CME pathway to enter cells.

CavME is a ligand-triggered process characterized by dependencies on cholesterol, lipid rafts, Dynamin, and a complex signaling pathway that is stimulated by tyrosine kinases and phosphatases [10]. The Rift Valley fever virus [11], simian virus 40 (SV40) [12], echovirus 1 [13], and respiratory syncytial virus (RSV) [14] all reportedly enter cell via caveolae. Dynamin is a GTPase protein essential for membrane fission during CME and CavME. Three dynamin-encoding genes (Dynamin I, II, and III) have been identified [15, 16]. Dynamin I and III are mainly expressed in the brain, while Dynamin II is expressed ubiquitously [17].

Macropinocytosis as a direct endocytic route used to invade host cells by vaccinia virus [18, 19], HIV-1 [20], influenza A virus [21, 22], African swine fever virus (ASFV) [23], Nipah virus [24], papillomavirus type 16 [25], and RSV [26]. Macropinocytosis involves actin rearrangement, which ultimately leads to the development of numerous irregular ruffles and blebs on the cell membrane [27]. Other proteins involved in macropinocytosis include Ras-related C3 botulinum toxin substrate 1 (Rac1), Cdc42, Na^+^/H^+^ exchangers (NHEs), p21-activated kinase 1 (Pak1), phosphoinositide 3-kinase [PI(3)K], protein kinase C (PKC) and dynamin. The requirement for these factors varies with cell or virus types [28].

Rab GTPases play a fundamental role in the regulation of membrane traffic. Among them, Rab5 and Rab7 are involved in the endocytic process [29]. The three Rab5 isoforms (Rab5a, Rab5b and Rab5c) have all been implicated in regulation of the plasma membrane early during endosomes transport. [30] Rab5a is ubiquitously expressed, and reportedly contributes to the regulation of trafficking during the early endocytic pathway [31]. Rab7 (also known as Rab7a) is a late endosome-/lysosome-associated small GTPase [32]. Rab5a localizes to early endosomes, where it is involved in recruiting Rab7a and the maturation of these compartments to late endosomes [33]. Overexpression of wild-type or mutant Rab proteins with impaired ability to hydrolyze GTP increases the internalization rate of endocytosis, while expression of dominant negative mutants with impaired ability to bind GTP slows the kinetics of endocytosis [30].

To gain further insight into the molecular mechanisms underlying NDV entry, we used Western blotting, immunofluorescence and infectivity virus titer assays to quantify infectivity levels following treatment with specific inhibitors. Our findings indicate that NDV exploits the macropinocytosis and CME pathways to enter DF1 cells. Moreover, NDV particles are trafficked to endosomal structures in a Rab5a-dependent manner and profit from early endosomal acidification to efficiently infect DF1 cells.

## RESULTS

### NDV internalized by DF-1 cells undergoes replication

**Figure 1:**
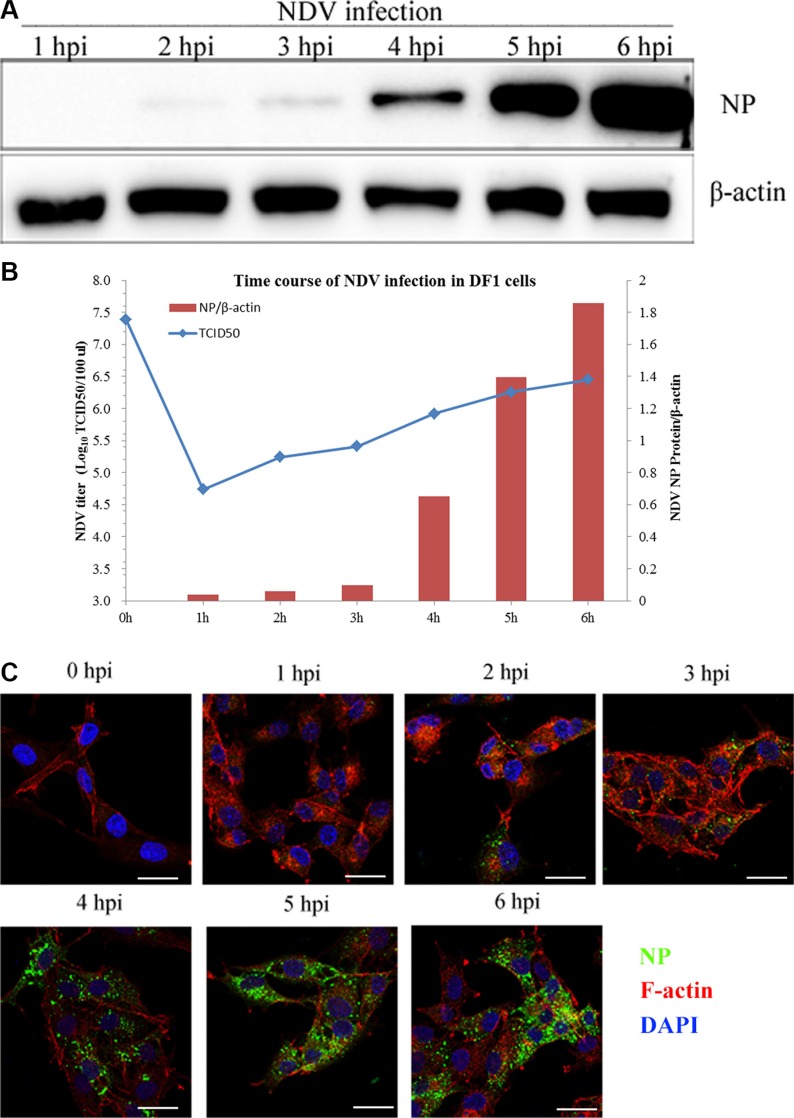
NDV internalization and propagation in DF-1 cells (**A**) Western blotting assay of NP expression in DF-1 cells at the indicated time points (1–6 h). b-actin was used as a sample loading control. (**B**) Supernatants from NDV-infected DF-1 cells collected 1 to 6 hpi. NDV titers were determined using TCID_50_ assays and depicted as a blue line on the graph. The NP/β-actin ratio is shown in brown red in the graph. (**C**) Time course of NDV infection in DF-1 cells. The cells were fixed at the indicated times and processed for Actin filaments (red), NP (green) and cell nucleus (blue), respectively. Scale bar = 20 μm.

DF-1 cells are a sensitive model for studying NDV. Using western blotting after exposing cells to 5 MOI NDV, viral NP proteins first detected 2 h after viral infection, and viral replication was shown to be time-dependent (Figure 1A). The viral titer in the supernatant from NDVinfected DF-1 cells gradually increased from 4.74 log_10_TCID_50_/100 μL at 1 hpi to 6.45 log_10_TCID_50_/100 μL at 6 h. At 0 hpi, the NDV titer was 7.38 log_10_TCID_50_/100 μL, which is very high and attributable to unbound NDVs present in the DF-1 supernatant (Figure 1B). Using CSL and immunofluorescent labeling of NP, we observed that internalized NDV was initially distributed to the cell margins. Later, however, the green fluorescent NP signal was observed in the cytoplasm (Figure 1C). These results indicate that NDV proteins and genomic RNA were probably produced within 2 hpi and completed the replicative cycle within 4 hpi. In subsequent experiments, therefore, 4 hpi was considered as the optimal time point for determining viral titers and protein expression levels.

### Role of clathrin-mediated pathways in NDV internalization

**Figure 2:**
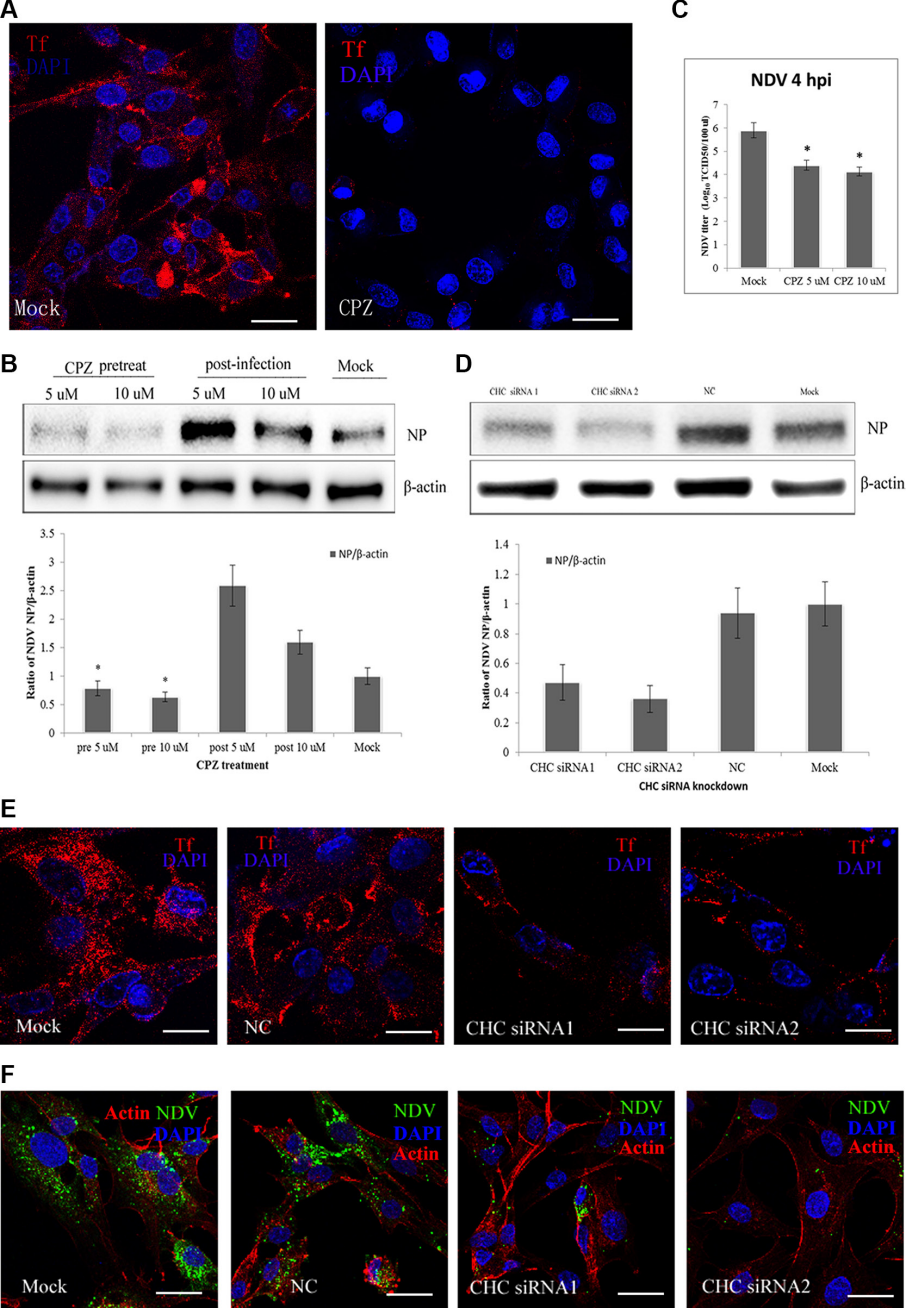
CME pathways involved in NDV entry into DF-1 cells (**A**) The CME inhibitor CPZ blocks transferrin entry. DF-1 cells were pretreated with CPZ and then fixed and incubated with TRITC-transferrin and DAPI. (**B**) CPZ inhibits NDV entry in a manner. DF1 cells were pretreated with CPZ, or CPZ was added 60 min after NDV infection. Western blotting was used to detect expression levels of the NDV proteins. Fold induction was determined by densitometry and analyzed using ImageJ software. (**C**) The NDV titers in the supernatants of Mock- or CPZ-treated cells were determined using TCID_50_ assays. The results are presented as the mean ± SD. *P < 0.05. (**D**) The efficiency of CHC siRNAs was analyzed by Western blotting. (**E**) The effect of siRNAs on transferrin uptake was observed using CLSM: TRITC-transferrin (red), cell nucleus (blue). (**F)** NDV entry was markedly suppressed in CHC siRNA-transfected DF-1 cells. Actin filaments (red), NP protein (green), cell nucleus (blue). Scale bar = 20 μm.

To assess the role of CME in NDV entry into DF-1 cells, the effect on virus entry of CPZ, which disrupts the assembly of clathrin lattices at the cell surface, was determined [34, 35]. Transferrin, a model molecule for the CME pathway, was internalized as a positive control. CPZ significantly decreased the uptake of transferrin (Figure 2A), confirming that it effectively blocked CME. Western blotting then demonstrated that pretreating DF-1 cells with CPZ significantly decreased NDV internalization. However, for NDVs that did enter DF-1 cells, CPZ enhanced NDV infection (Figure 2B), TCID_50_ assays indicated that CPZ inhibited NDV propagation (Figure 2C). To further evaluate the role of clathrin on NDV internalization, its expression in DF-1 cells was knocked down using CHC-specific siRNAs and confirmed by western blotting (Figure 2D). Compared to control siRNA, CHC siRNA1 and siRNA2 both significantly inhibited transferrin uptake (Figure 2E). Likewise, NDV internalization was greatly inhibited after CHC knockdown (Figure 2F). These findings suggest that entry of NDV into DF-1 cells is dependent on CME.

### The NDV endocytic pathway does not require cholesterol but is dependent on dynamin

**Figure 3:**
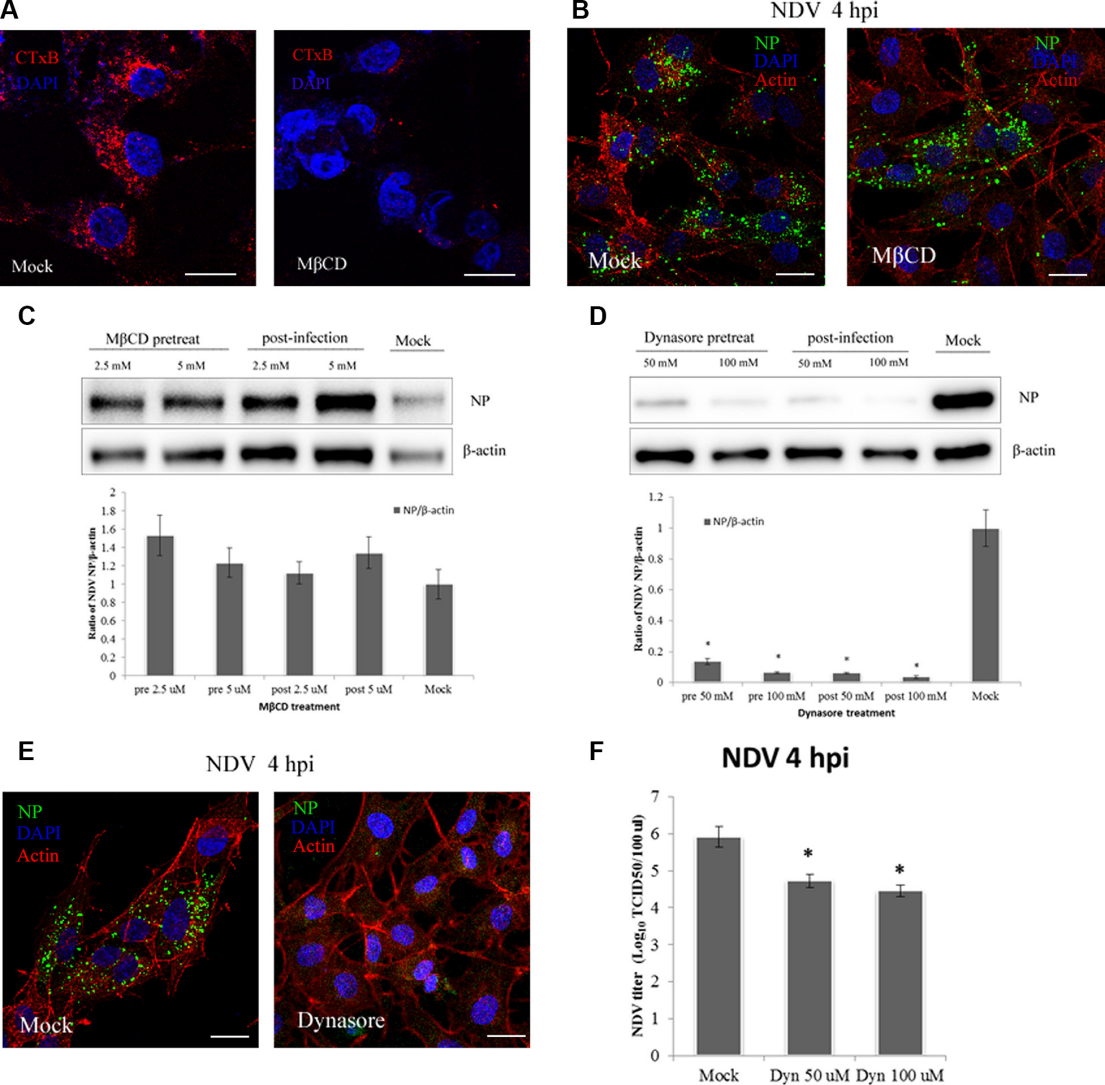
NDV entry into DF1 cells is dynamin-dependent, whereas plasma membrane cholesterol is dispensable (**A**–**B**) CLSM imaging revealed the inhibitory effect of MbCD on CTxB uptake (red; upper left panels), whereas NDV uptake was not effectively blocked (upper right panels). (**C**–**D**) Western blotting showing inhibition of NDV entry by MbCD or dynasore. The ratio of NP/b-actin is shown. (**E**) Dynasore inhibition of NDV entry observed using CLSM: actin filaments (red), NP protein (green), cell nucleus (blue). (**F**) NDV titers in the supernatant of Mock- or dynasore-treated cells were determined using the TCID_50_ assays. The results are presented as the mean ± SD, *P < 0.05. Scale bar = 20 μm.

Previous studies showed that NDV recognizes ganglioside receptors on the cell surface and enters COS-7 cells via CavME [3], which is strictly dependent on cholesterol, the main component of lipid rafts within caveolae. To evaluate the role of CavME in NDV entry into DF-1 cells, we tested the effect of MbCD, which extracts cholesterol from the plasma membrane. CTxB, which utilizes CavME to enter cells, was administered as a positive control. CLSM imaging showed that MbCD effectively inhibited the internalization of CTxB by DF-1 cells (Figure 3A). By contrast, images acquired at 4 hpi indicated that MbCD had little effect on NDV internalization as compared to the mock control (Figure 3B). Western blotting confirmed the absence of an inhibitory effect of MbCD; instead, viral replication was increased at 4 hpi (Figure 3C). Thus CavME does not appear to mediate NDV entry into DF-1 cells.

GTPase dynamin mediates the dissociation of newly formed endocytic vesicles from the cell membrane, and is an important regulator of both CME and CavME [36]. To test the effects of dynamin on NDV infection at 4 hpi, dynasore, a reversible dynamin inhibitor [37], was used to treat DF-1 cells. Western blotting indicated that dynasore applied before or after NDV application dose-dependently inhibited DF-1 cell infection by NDV (Figure 3D). CLSM also revealed that dynasore significantly inhibited NDV internalization, and viral factories disappeared in dynasore-treated DF-1 cells (Figure 3E). Furthermore, TCID_50_ assay showed that dynasore dose-dependently decreased virus production (Figure 3F). These observations indicate that both NDV virus entry and post-entry processing are dynamin-dependent in DF-1 cells.

### NDV entry is dependent on the actin rearrangement process

**Figure 4:**
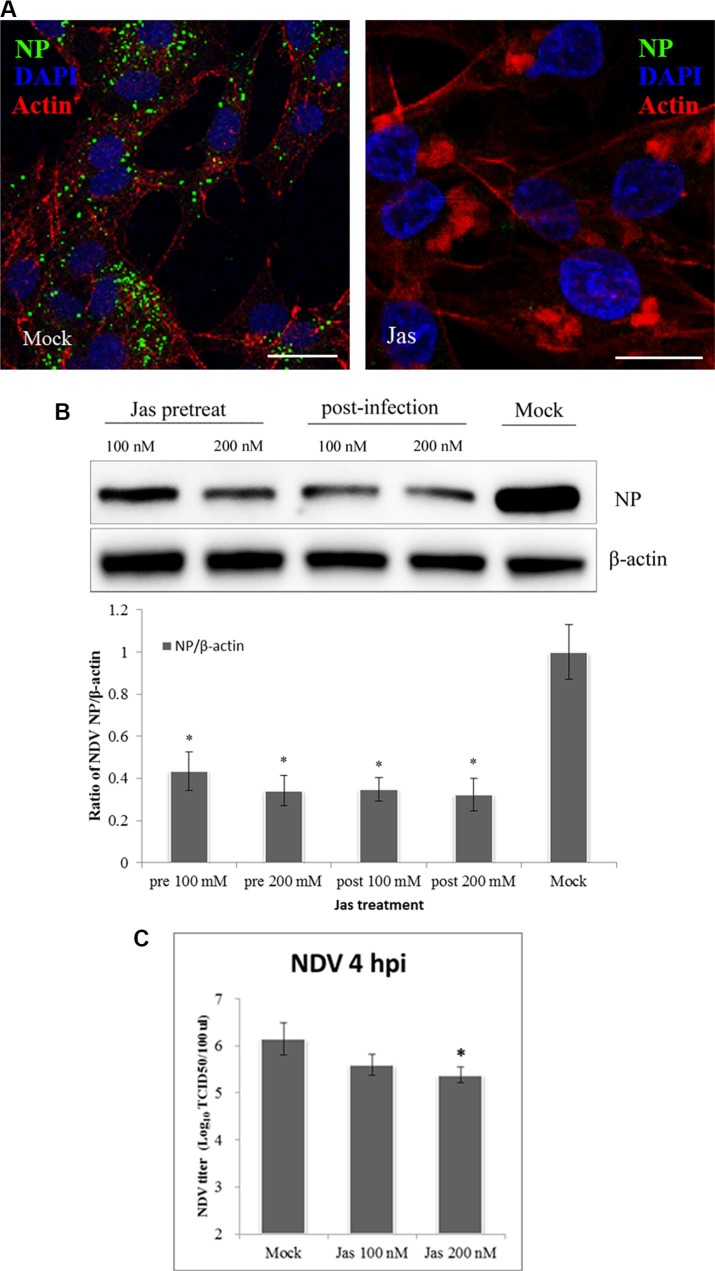
Actin rearrangements during NDV internalization (**A**) Jasplakinolide inhibition of NDV entry into DF-1 cells. Pretreated cells (200 nM jasplakinolide) were infected with 5 MOI NDV for 4 h at 37°C and observed using CLSM: Actin filaments (red), NP (green), and cell nucleus (blue). (**B**) Western blotting showing jasplakinolide inhibition of NDV entry as indicated by NP levels. The ratio of NP/b-actin is shown. (**C**) The NDV titers of the supernatant of Mock- and jasplakinolide-treated cells were determined using TCID_50_ assays. The results are presented as the mean ± SD, *P < 0.05, Scale bar = 20 μm.

Recent studies have demonstrated that the actin cytoskeleton is involved in the regulation of macropinocytosis [38], during which actin polymerization and cytoskeletal rearrangement produce outward protrusions in the plasma membrane, including lamellipodia, circular ruffles or blebs [39, 40]. To determine whether NDV entry into cells depends on actin, we used jasplakinolide, which stabilizes actin polymers and prevents actin rearrangement [41]. After pretreating DF-1 cells with jasplakinolide for 60 min, the cells were infected with NDV in the continued presence of jasplakinolide. In a subsequent CLSM analysis the amount of NDV in the jasplakinolide-treated cells was significantly lower than in Mock-treated cells (Figure 4A). These suggests NDV entry into cells was inhibited by suppressing actin dynamics. This was confirmed by western blotting, which showed that jasplakinolide added before or after NDV application dose-dependently reduced NDV infection of DF-1 cells (Figure 4B). In addition, TCID_50_ assays showed that jasplakinolide reduced both virus entry and titer relative to that observed in untreated cells (Figure 4C). These findings indicate that macropinocytosis may be another pathway for productive entry of NDV.

### NDV entry depends on NHE

**Figure 5:**
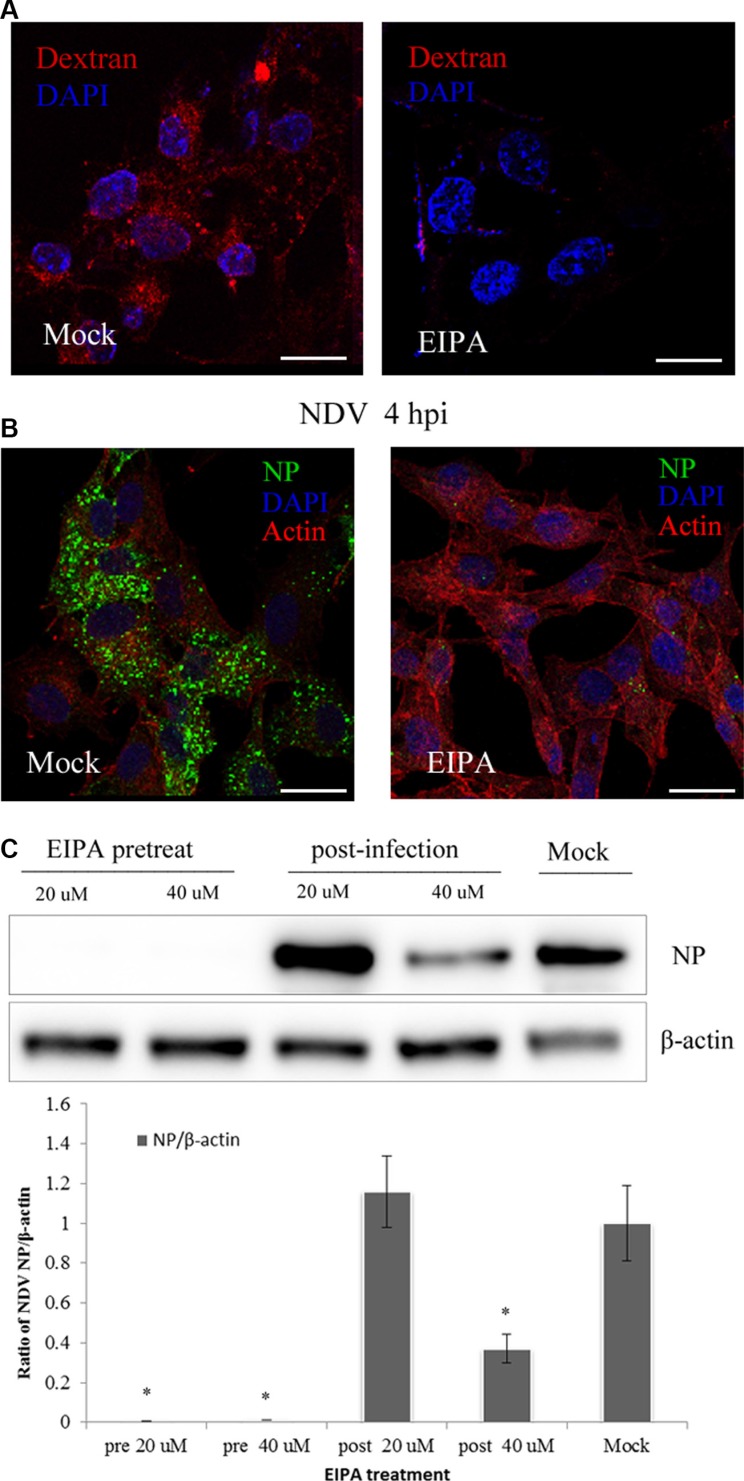
NDV entry involves macropinocytosis (**A**–**B**) Inhibition of dextran uptake into DF-1 cells by EIPA. Pretreating cells with 40 μM EIPA resulted in near-complete blockade of NDV uptake. (A) Dextran (red), cell nucleus (blue). (B) Actin filaments (red), NP protein (green), cell nucleus (blue). (**C**) Western blotting showing EIPA inhibition of NDV entry. The ratio of NP/b-actin is shown. *P < 0.05. Scale bar = 20 μm

Macropinosome formation reportedly requires NHE activity, which has become a diagnostic criterion for macropinocytosis [42]. We assessed the role of macropinocytosis in NDV infection using EIPA, a specific inhibitor of NHE [43]. Activation of macropinocytosis can induce a short-lived elevation of nonspecific fluid uptake. To test the ability of EIPA in inhibit fluid uptake, DF-1 cells were pretreated with EIPA for 60 min and then incubated for 45 min with the well-established fluid-phase endocytic marker, dextran-Texas red. Subsequent CLSM demonstrated that dextran uptake was almost completely inhibited by EIPA (Figure 5A). When DF-1 cells pretreated for 60 min with EIPA were infected with 5 MOI NDV, the amount of NDV taken up was markedly decreased, and no viral factories were observed (Figure 5B). By contrast, when cell were treated with EIPA 60 min after NDV entry, its ability to prevent NDV propagation was significantly decreased (Figure 5C). These findings further confirm that NDV utilizes macropinocytosis for internalization into DF-1 cells.

### NDV entry is independent for PI(3)K but dependent on PKC activation

**Figure 6:**
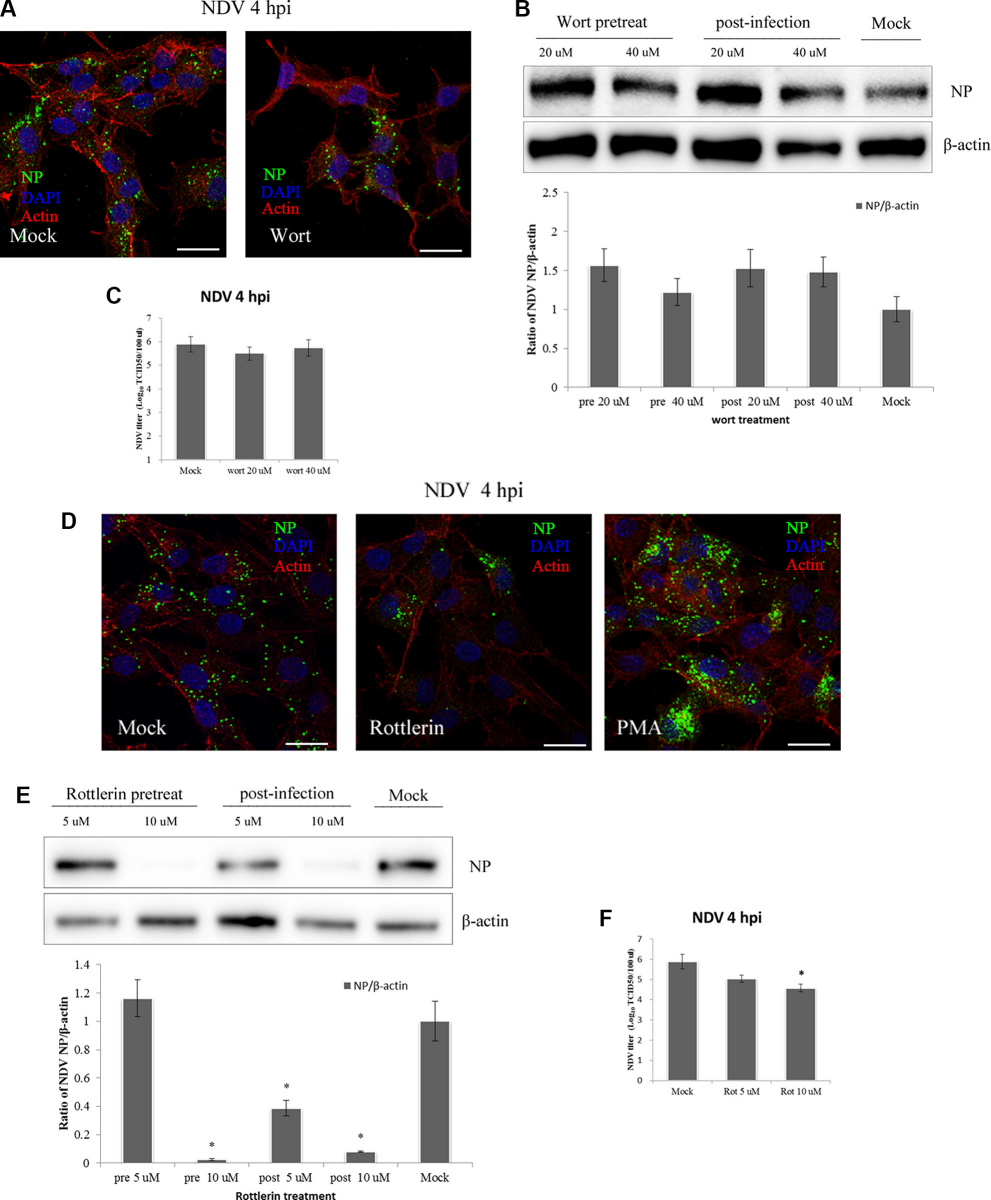
PKC, not PI(3)K, is required for NDV entry and propagation in DF-1 cells (**A**) DF-1 cells were pretreated with 40 μM wortmannin and infected 5 MOI NDV for 4 h. Cells were fixed and observed using CLSM: Actin filaments (red), NP (green) and cell nucleus (blue). (**B**) DF-1 cells were treated with wortmannin (20 μM or 40 μM) for 4 h before or after NDV infection, and cells lysates were prepared for Western blot analysis. The ratio of NP/b-actin is shown. (**C**) The NDV titers in the supernatants of Mock- or wortmannin-treated cells were determined using TCID_50_ assays. (**D**) Pretreated cells (10 μM rottlerin or PMA) were infected with 5 MOI NDV for 4 h at 37°C and observed using CLSM as A. (**E**) DF-1 cells were treated with rottlerin (5 μM or 10 μM) for 4 h before or after NDV infection, and cells lysates were prepared for western blot analysis as in B. (**F**) NDV titers of the supernatants of Mock- and rottlerin-treated cells were determined using the TCID_50_ assays. The results were presented as the mean ± SD. *P < 0.05. Scale bar = 20 μm.

Protein kinase C (PKC) is a Ca^2+^ and diacylglycerol-dependent serine/threonine kinase. It is activated by RTKs or PI(3)K, and after dissociation from the plasma membrane, it promotes ruffling and macropinosome formation [28]. PI(3)K participates in several phases of macropinocytosis, including membrane ruffling, closure, and trafficking and fusion of macropinosomes [44, 45]. PKC activators such as phorbol-12-myristate-13-acetate (PMA) induce ruffling and fluid uptake in the absence of receptor activation [28, 46]. When DF-1 cells were pre-treated with 40 μM wortmannin, a inhibitor of PI(3)K, we detected no inhibition of NDV entry using CLSM (Figure 6A). This was confirmed by western blotting, which showed no inhibition of NDV entry by wortmannin, whether administered before or after NDV infection. TCID_50_ assays also showed that wortmannin had little if any effect on NDV entry (Figure 6C). Interestingly, wortmannin administration appeared to promote NDV infection (Figure 6B).

To further examine the role of PKC, NDV-infected DF-1 cells were pre-treated with rottlerin and PMA, respectively. CLSM revealed that at the indicated time points, rottlerin effectively inhibited NDV internalization, and the number of viral factories was decreased in rottlerin-treated cells. By contrast, NDV entry was increased in PMA-treated cells compared to untreated cells (Figure 6D). Similarly, western blotting showed that rottlerin dose-dependently decreased viral production, and that application of 10 mM rottlerin completely inhibited NDV replication. Administration of rottlerin after NDV infection also decreased the amount of viral protein secreted (Figure 6E), and TCID_50_ assays demonstrated that NDV entry was effectively blocked by rottlerin (Figure 6F). PKC thus appears to be a critical mediator of NDV entry into DF-1 cells.

### The Rac1-Pak1 pathway is involved in NDV entry

Macropinocytosis depends on several kinases, including Rac1 and Pak1. Activated Rac1 improves actin polymerization at the cell membrane, while Pak1 is a Rac1-activated serine/threonine kinase [27] that governs cytoskeletal dynamics and motility and is required at all stages of macropinocytosis [28].

**Figure 7:**
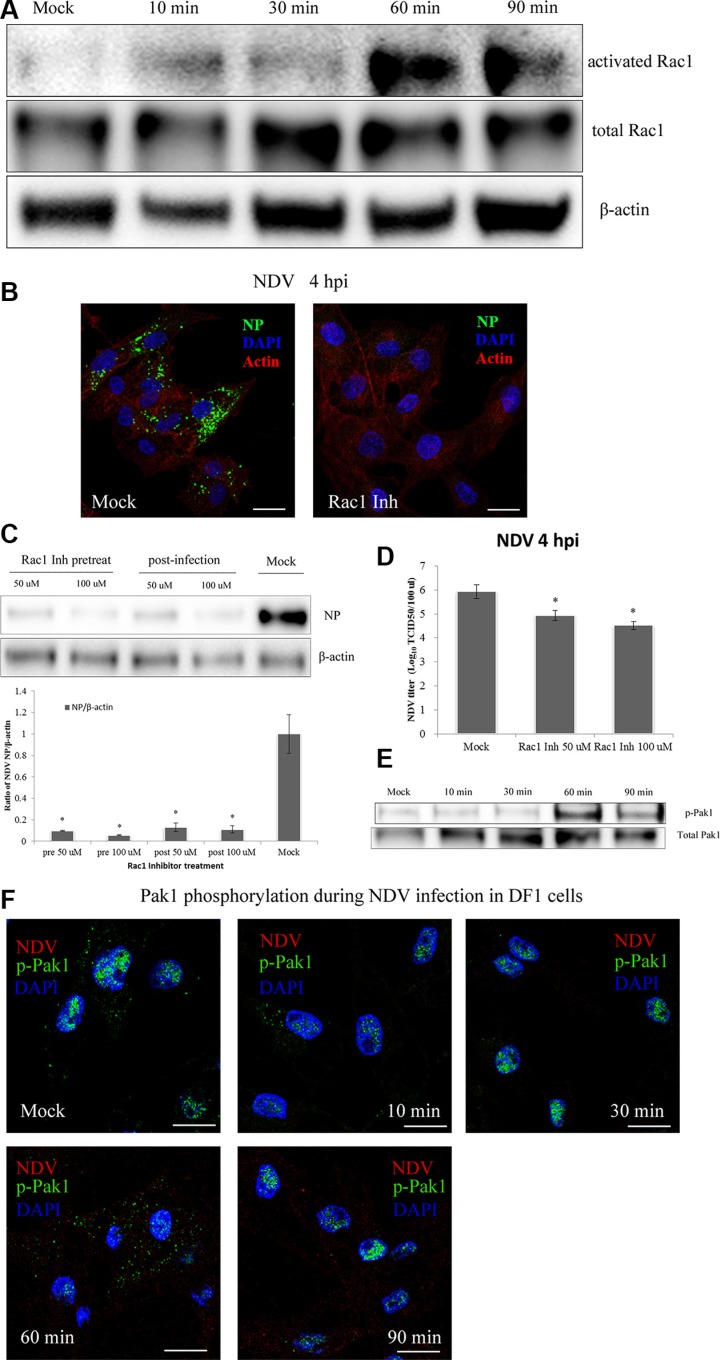
NDV entry into DF-1 cells involves Rac1 activation and Pak1 phosphorylation (**A**) Activation of Rac1 during NDV entry. DF-1 cells were infected with 5 MOI NDV, and Rac1 activation was measured using GST-Pak1-PBD pull-down assays. (**B**) Rac1 inhibition prevented NDV entry. Pretreated cells (100 μM Rac1 inhibitor) were infected for 4 h at 37°C and observed using CLSM: Actin filaments (red), NP (green), and cell nucleus (blue). (**C**) Western blots showing that treatment with a Rac1 inhibitor for 4 h before or after infection inhibited NDV entry into DF-1 cells. (**D**) NDV titers of supernatants of Mock- or Rac1 inhibitor-treated cells were determined by using TCID_50_ assays. The results are presented as the mean ± SD. *P < 0.05. (**E**) NDV infection led to Pak1 phosphorylation early after infection. Cells were infected (5 MOI), after which levels of phosphorylated Pak1 (Ser144) and total Pak1 at the indicated times were determined using western blotting. (**F**) CLSM imaging showing the distribution of p-Pak1 in DF-1 cells at the indicated after infection with 5 MOI NDV. Scale bar = 20 μm.

To examine the role of Rac1 on NDV entry into DF-1 cells, the activated form of Rac1 bound to GST-Pak1-PBD beads was applied to a GST pull-down test. Western blotting was then used to detect changes in the levels of activated Rac1 at indicated times during NDV entry. The results showed a slight increase in activated Rac1 10 min after NDV entry, as compared to untreated DF-1 cells. At 30 min post-infection (mpi), the level of activated Rac1 the same or lower than at the 10 min time point. At 60 mpi, however, the activation level rapidly increased and reached a peak 5-fold higher than control. The level of activated Rac1 then decreased and was half the peak level at 90 mpi (Figure 7A). NDV invasion thus induces Rac1 activation, which occurs mainly during NDV entry.

The contribution of Rac1 to NDV infection was examined using a specific Rac1 inhibitor. Using CLSM we observed that pretreating DF-1 cells with a Rac1 inhibitor greatly decreased NDV entry completely eliminated viral factories in the cells (Figure 7B). Western blotting confirmed that pretreatment using a Rac1 inhibitor effectively prevented NDV entry into DF1 cells. In addition, treating the cells with a Rac1 inhibitor 1 hpi inhibited the synthesis of viral proteins (Figure 7C). These findings were consistent with the results of TCID_50_ assays (Figure 7D) and indicate that Rac1 activation is a crucial step in the entry and propagation of NDV.

To test whether NDV infection also activates Pak1, DF-1 cells were serum-starved for 1 h and then infected with 5 MOI NDV for 10 min, 30 min, 60 min or 90 min. Subsequent western blotting showed that Pak1 phosphorylation (S144) rapidly increased after NDV infection, reaching a peak 60 mpi and decreasing at 90 mpi (Figure 7E). Immunofluorescent labeling indicated that the p-Pak1 signal was weaker in control cells and accumulated in the nucleus. At 60 mpi, the p-Pak1 signal reached their highest intensity and surrounded the nucleus. The signal then weakened until 90 mpi (Figure 7F). This result coincides with the results of the western blotting and suggests the Rac1-Pak1 pathway is involved in NDV entry.

### Intracellular trafficking of NDV employs Rab5a-positive vesicles

**Figure 8:**
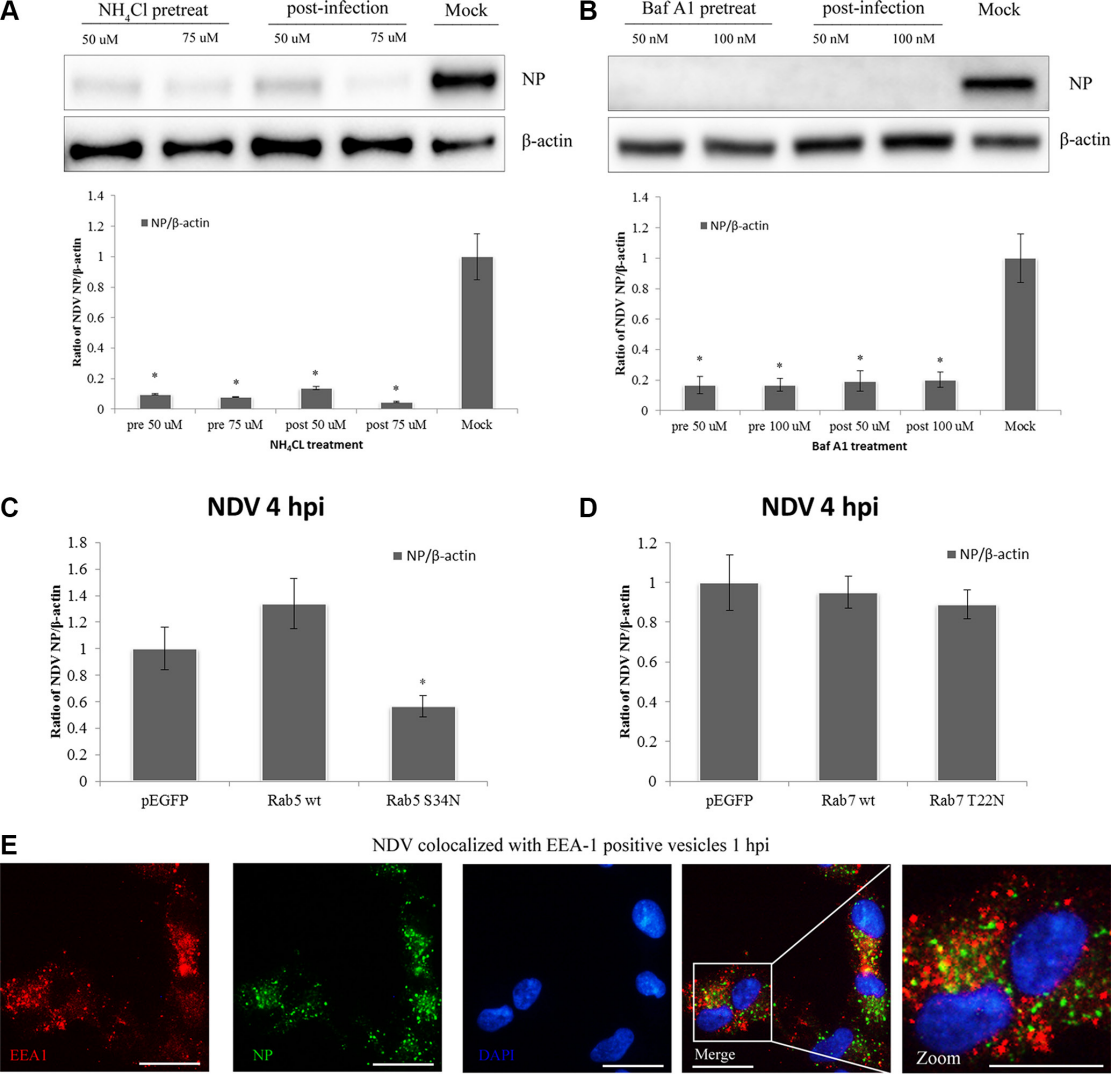
NDV infection is pH-dependent and intracellular trafficking involves Rab5a-positive early endosomes (**A**–**B**) DF1 cells were treated with NH_4_Cl or Baf A1 for 4 h before or after NDV infection, and cells lysates were prepared for western blot analysis. The NP/b-actin ratio is shown. (**C**) Intracellular trafficking of NDV requires Rab5a. DF-1 cells were transfected with pEGFP, pEGFP-Rab5wt or pEGFP-Rab5S34N, infected with 5 MOI NDV for 4 h at 37°C and analyzed by western blotting. The NP/b-actin ratio is shown. *P < 0.05. (**D**) Rab7a function is dispensable for NDV intracellular trafficking. DF-1 cells were transfected with pEGFP, pEGFP-Rab7wt or pEGFP-Rab7T22N, and analyzed using Western blotting as in C. (**E**) NP co-localized with early endosome marker EEA-1-positive vesicles. DF-1 cells were infected with 5 MOI NDV for 1 h and observed using CLSM: EEA1 (red), NP (green), and cell nucleus (blue). Scale bar = 20 μm.

The experiments summarized above indicate that NDV entry into DF-1 cells involves macropinocytosis. It has been previously reported that macropinosomal maturation requires intravesicular acidification and acquisition of Rab5a, which is followed by recruitment of Rab7 prior to endolysosomal transition [47]. We therefore tested whether NDV infection is pH-dependent using the weak base NH_4_Cl, which neutralizes intravesicular pH, and Baf A1, which specifically inhibits the H+-ATPase responsible for intravesicular acidification [48, 49]. Subsequent western blotting indicated that both NH_4_Cl and Baf A1 greatly impaired the ability of NDV to infect DF-1 cells (Figure 8A–8B).

A number of Rab GTPases are involved in the regulation of secretory and endocytic membrane trafficking pathways [50]. Rab5a is in charge of the homotypic tethering and fusion of early endosomes [51], while Rab7a is more specifically related to degradative compartments such as late endosomes and lysosomes [52]. We therefore investigated the roles of Rab5a and Rab7a in NDV infection using DF-1 cells transiently transfected for 24 h with pEGFP control, pEGFP-Rab5wt and pEGFP-Rab5S34N. The transfectants were then infected with 5 MOI NDV for another 4 h, and the level of NP protein expression was determined. Western blotting indicated that overexpression of wild-type Rab5a (Rab5wt) significantly increased NDV infection as compared to GFP vector control, whereas overexpressing a DN Rab5 mutant (Rab5S34N) significantly inhibited NP expression as compared to Rab5wt and the GFP vector control (Figure 8C). In addition, CLSM imaging revealed that EEA1-positive compartments co-localized with NP at 1 hpi (Figure 8D). This indicates that EEA1-positive early endosome compartments and Rab5a important during NDV intracellular trafficking.

The role of Rab7a in NDV infection was examined using DF-1 cells transiently transfected with pEGFP, pEGFP-Rab7wt or the DN mutant pEGFP-Rab7T22N. The percentage of transfected-infected cells was determined as earlier described. The DN form of Rab7a prevents transport from early to late endosomes [53]. Western blotting analysis showed the overexpression of Rab7T22N and Rab7wt in DF-1 cells had no effect on NDV infection (Figure 8E). The macropinosomes mediating NDV trafficking are possibly dependent on Rab5apositive vesicles but not Rab 7a-positive ones.

**Figure 9:**
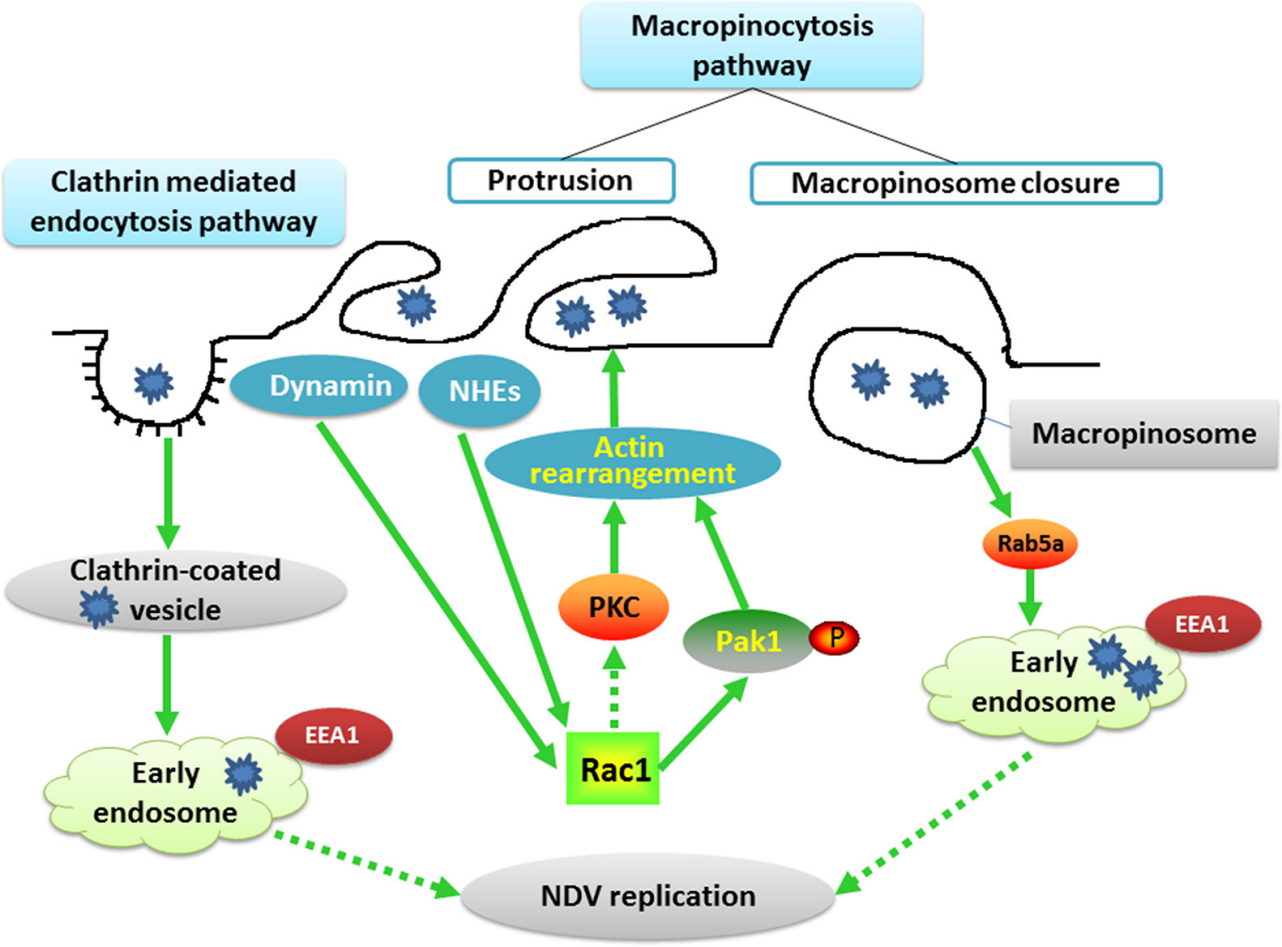
Schematic illustrating NDV entry into DF1 cells The schematic depicts NDV entry into DF1 cells through macropinocytosis and CME. The binding of NDV activates NHEs, dynamin, Rac1, Pak1 and PKC, which in turn triggers actin rearrangement and plasma membrane ruffling. These dynamic protrusions provide the membrane and energy required for formation of endocytic vacuoles. After macropinosome closure, the internalized NDV is trafficked to early endosomes in a Rab5a-dependent process. NDV binding also induces virus internalization via CME. The internalized vesicles are delivered to early endosomes, and the acidic pH of the endosomes trigger viral propagation.

Altogether, our data indicate that early endosome acidification is required for NDV infection and that internalized virions are transported toward Rab5a-positive endosomal vesicles, where a functional GTPase Rab5a is critical for progression of the NDV replication cycle. The proteins and signaling pathways involved in NDV entry into DF-1 cells are shown in Figure 9.

## DISCUSSION

In the present study, we investigated whether DF-1 cells utilize an endocytic mechanism for NDV uptake. The pharmacological inhibitors MbCD, CPZ and dynasore were used to assess the involvement of CavME and CME. Western blotting demonstrated that specific blockade of CME reduced NDV production by approximately 40%. By contrast, specific blockade of CavME had little effect on NDV infection. These results indicate that NDV enters cells via CME, but not CavME. However, CME is not the only endocytic pathway employed by NDV. We also observed that NDV continues to infect DF-1 cells after CME has been blocked. These findings are not in agreement with those of Cantín *et al*. [3]. It thus seems likely that alternative endocytic pathways are used by NDV.

Previous reports have demonstrated that some paramyxoviridae, such as RSV, Nipah virus and human metapneumovirus, employ macropinocytosis to invade host cells [24, 26, 58]. We therefore tested the possibility that some NDV entry is mediated by macropinocytosis. Our results indicate that NDV entry into DF-1 cells is sensitive to inhibitors of Rac1, Pak1 and NHE. Rac1 is an important signaling factor that activates its specific effector, Pak1, to induce actin rearrangements that in turn facilitate formation of lamellipodia and circular ruffles [59, 60]. As a downstream target protein of Rac1, Pak1 is involved in actin cytoskeleton dynamics and all phases of macropinocytosis [27, 61]. NDV entry into DF-1 cells causes a rapid activation of Rac1 that reaches a peak within 60 min, and Rac1 inhibiting significantly reduces NDV infection. These findings indicate that Rac1 plays a crucial role in the NDV entry process. Moreover, NDV-infected cells exhibited rapid Pak1phosphorylation, after which the activated p-Pak1 accumulated near the nucleus. Thus entry of NDV into DF-1 cells requires activation of the Rac1-Pak1 signaling pathway.

PI(3)K and its effectors are responsible for the formation of the lipid microdomain ruffles and macropinocytic cups that serve as platforms for signaling and cytoskeletal modulation. Activated by Rac1, PI(3)K is essential for closure of macropinocytic cups and, possibly, ruffle formation and macropinosome fusion [28]. We found that NDV infection was little affected by inhibited by PI(3)K inhibition using wortmannin. Indeed, the addition of a high concentration of wortmannin after NDV entry promoted NDV infection. These results indicate that other kinases give rise to macropinocytosis in DF-1 cells. Given the importance of PKC to macropinocytosis, we tested the effect of the PKC inhibitor rottlerin and the known PKC activator PMA on the internalization of NDV by DF-1 cells. Our results indicate that PKC activation or deactivation markedly affects NDV entry and propagation. These findings indicate that NDV entry into DF-1 cells involves several activities characteristic of macropinocytosis.

Using CLSM, we observed that the early endosomal vesicle marker EEA1 colocalizes with NDV NP wrapped in macropinosomes, which had detached from the plasma membrane. This suggests that after their formation macropinosomes may acquire an EEA1-labeled protein, or they may have rapidly fused with EEA1-labeled vesicles, which is consistent with an earlier report [2]. Overexpression of a DN Rab5a mutant inhibited NDV infection, indicating NDV infection is dependent on passage through Rab5a-containing early macropinosomes. On the other hand, a DN Rab7 inhibition of vesicle maturity had no effect on viral infection, indicating NDV penetration does not involve Rab7-positive vesicles. This observation was in agreement with previous reports suggesting that early macropinosomes undergo acidification and that NDV is susceptible to acidic pH [2].

In sum, this comprehensive analysis of the endocytic pathway of NDV demonstrated for the first time that NDV utilizes macropinocytosis and CME to enter DF-1 cells, though macropinocytosis is the primary pathway employed by NDV prior to CME. The evidence for NDV usage of macropinocytosis provides additional insight into the mechanism underlying viral entry and propagation. Moreover, the components of host cells involved in this pathway may serve as potential targets for tumor cell therapy. This work may be also used as a reference for the prevention and control of NDV and other paramyxoviruses.

## MATERIALS AND METHODS

### Cells and virus

The NDV velogenic strain ZJ1 was isolated and identified in the Key Laboratory of Animal Infectious Diseases, Yangzhou University [54]. The virus was propagated in embryonated chicken eggs and titrated onto DF-1 cells to determine the tissue culture infective dose (TCID_50_) using the Reed and Muench method [55]. The galline embryonic fibroblast cell line (DF-1) was purchased from American Type Culture Collection (ATCC, Manassas, VA, USA) and maintained in Dulbecco's modified Eagle's medium (DMEM) supplemented with 10% fetal bovine serum, 0.1 mg/mL streptomycin, and 100 U/mL penicillin at 37°C in 5% CO_2_.

### Inhibitors, antibodies and reagents

Pharmacological inhibitors and control reagent dimethyl sulfoxide (DMSO) were purchased from Sigma-Aldrich (St. Louis, MO, USA) and used at appropriate concentrations that maintained cell vitality (Table 1). RIPA buffer, streptomycin, penicillin, monoclonal anti-EEA1 antibody, tetramethylrhodamine-phalloidin (TRITC-phalloidin), mouse anti-β-actin monoclonal antibody (mAb), horseradish peroxidase-conjugated (HRP) goat anti-rabbit, and goat anti-mouse IgG antibody were purchased from Sigma-Aldrich. SDS-PAGE sample loading buffer and 4,›6-diamidino-2-phenylindole (DAPI) were from Beyotime (Nantong, China). Lipofectamine^®^ Plus^TM^ transfection reagent, fetal bovine serum (FBS), DMEM, enhanced chemiluminescent reagent (ECL), TRITC-transferrin, Alexa Fluor^®^ 594 conjugate cholera toxin subunit B (CTxB), and Texas red-dextran, and Alexa Fluor^®^ 488 goat anti-mouse secondary antibody were from Thermo Fisher Scientific (Waltham, MA, USA). The Active Rac1 detection kit was from the Cell Signaling Technology (Danvers, MA, USA). The mAb against NDV nucleoprotein (NP; Herts/33 strain) was prepared in our laboratory. The primary antibody against phospho-Pak1 (phospho-S144) and anti-Pak1 was from Abcam (Cambridge, MA, USA).

**Table 1:** Inhibitors and effect on cell

Inhibitor	Working concentration	Effect on cell
Chlorpromazine (CPZ)	5 μM or 10 μM	Inhibitor of clathrin-mediated endocytosis
Methyl-β-cyclodextrin (MβCD)	2.5 mM or 5 mM	Disrupt lipid rafts in cells by depleting the cholesterol component
Dynasore	50 μM or 100 μM	Inhibitor of dynamin
5-(N-methyl-N-isobutyl) amiloride (EIPA)	20 μM or 40 μM	Inhibitor of the NHEs
Jasplakinolide	100 nM or 200 nM	Induce actin polymerization and prevent the actin rearrangement
Rottlerin	5 μM or 10 μM	PKC inhibitor
Phorbol-12-myristate-13-acetate (PMA)	5 μM or 10 μM	PKC activator
wortmannin	20 μM or 40 μM	Inhibitor of the PI(3)K
Rac1 inhibitor	50 μM or 100 μM	Inhibitor of the Rac1 kinase
Ammonium chloride (NH_4_CL)	50 μM or 75 μM	Neutralizes intravesicular pH
Bafilomycin A1 (Baf A1)	50 nM or 100 nM	Inhibitor of endosomal acidification

### Pharmacological inhibitor and NDV infection

DF-1 cells were seeded into six-well plates at a density of 2 × 10^5^ cells per well 24 h prior to infection. Pharmacological inhibitors were dissolved in DMSO to selected concentrations, after which their effects on the viability of DF-1 cells exposed to NDV was tested. After pretreating DF-1 cells with an inhibitor at 37°C for 60 min, the cells were exposed to NDV at a multiplicity of infection (MOI) of 5 for 1 h at 4°C and then incubated for 1 h to 37°C before complete medium was added. Four hours post-infection (hpi), the cell supernatant and pellets were harvested, separately. Expression of NP was analyzed using western blotting, and NDV titers were determined using the TCID_50_ assay.

### Western blotting

Cells were lysed in RIPA buffer containing protease inhibitors, then cleared by centrifugation for 10 min at 12,000 *g* at 4°C. The lysates were further denatured by incubation for 5 min at 95°C in SDS-PAGE sample loading buffer. The samples were then separated on a 10% polyacrylamide gel (Bio-Rad, Hercules, CA, USA) and electrotransferred (250 mA for 90 min) to a nitrocellulose membrane, after which the membrane was blocked for 2 h in Tris-buffered saline (TBS) solution containing 0.1% Tween-20 (TBST) and 5% non-fat milk. The membrane was then incubated overnight at 4°C with mouse anti-NP monoclonal antibodies (1:2,000 dilution), washing three times with TBST, incubated for 2 h at room temperature with HRP-labeled anti-rabbit or anti-mouse IgG antibody (1:10,000 dilution), and washed again three times with TBST. The protein bands were developed using ECL reagent, visualized using a Tanon 5200 automatic chemiluminescence image analysis system (Tanon, Shanghai, China) and quantified using ImageJ software [56, 57].

### Short interfering RNA (siRNA)

SiRNA oligos targeted against *Gallus gallus* clathrin heavy chain (CHC) (Genbank Accession Number: NM_001080117.1) were designed and purchased from GenePharma Biotech (Shanghai, China). The specific sequences included CHC siRNA1 (chicken-1419): 5′- GCU AGC ACU UAG UGU CUA UTT-3′, CHC-siRNA2 (chicken-2430): 5′- CCG CCU ACC UGU UGU UAU UTT-3′, and non-targeting siRNA (negative control, NC) oligo: 5′-UUC UCC GAA CGU GUC ACG UTT-3′. The CHC siRNAs and NC siRNA (100 nmol/well) were transfected into 60% confluent DF-1 monolayers using Lipofectamine^®^ Plus^TM^ according to the manufacturer's instructions. Six hours post-transfection, the culture medium was replaced with DMEM supplemented with 1% FBS (v/v). After incubation for 24 h, the transfected cells were infected with NDV at an MOI of 5 and harvested at 4 hpi.

### Indirect immunofluorescence

DF-1 cells grown on coverslips overnight were infected with NDV at an MOI of 5. The cells were then fixed in 4% paraformaldehyde, permeabilized with 0.5% Triton X-100 for 10 min, blocked in PBS containing 3% BSA, and incubated first with mouse anti-NP Ab and then with Alexa Fluor^®^ 488- conjugated goat anti-mouse IgG. Cell nuclei were stained with DAPI (1:500 dilution) for 10 min and, sometimes, and monolayers were incubated with TRITC-phalloidin for 10 min at 37°C. The coverslips were then mounted on glass slides and visualized using a confocal laser scanning microscope (CLSM, Nikon Eclipse 80i; Nikon, Tokyo, Japan).

### Trf, Dx and CtB uptake assays

DF1 cells grown on coverslips were left untreated or pretreated with selected inhibitors and incubated for 45 min with 5 μg/mL transferrin, for 90 min with 10 μg/mL CTxB, or for 45 min with 1 mg/ml dextran, after which the cells were washed three times with PBS, followed by three 5-min acid washes (1.5 M citric acid PBS, pH 5.5) to remove any unbound reagent. In some experiments, DF-1 cells were pretreated for 45 min at 37°C with DMSO or pharmacological inhibitors and then infected with 5 MOI NDV for 1 h at 4°C before incubation with dextran, transferrin, or CTxB. Finally, the monolayers were mounted, and the status of the transferrin, CTxB, and dextran internalization was visualized using CLSM.

### TCID_50_ assay

The collected supernatants from NDV-infected cells were centrifuged to remove cell fragments. Serial 10-fold dilutions of the virus stock were prepared in serum-free DMEM and added into 96-well plates. Eight replicates were prepared for each gradient, and 100 μL of the virus diluent were added to each well. Thereafter, 100 μL of the DF-1 cell suspension in DMEM with 10% FBS were added to each well at a density of 1.5 × 10^6^ cells/mL and mixed. The cells were then incubated for about 60 h at 37°C under 5% CO_2_, and the number of wells with or without a cytopathic effect (CPE) was counted. TCID_50_/100 μL values were calculated using the Reed-Muench method [35].

### Plasmids and transfections

The open reading frame of wild-type (wt) Rab5a (Genbank accession number: NM_001006363.2), Rab7a (Genbank accession number: XM_003641978.3), and dominant-negative (DN) GTP-binding defective Rab5a (S34N) and Rab7a (T22N) gene sequences were artificially synthesized (Sangon Biotech, Shanghai, China) and separately subcloned into a pEGFP-C3 plasmid vector (Takara Biomedical Technology Co., Ltd. Dalian, China). The corresponding plasmids were then designated pEGFP-Rab5wt, pEGFP-Rab7wt, pEGFP-Rab5S34N and pEGFP-Rab7T22N, respectively. The plasmids were transfected to 80% confluent monolayers grown on coverslips using 2 μg of DNA and 6 mL of Lipofectamine^®^ Plus^TM^ following the manufacturer's recommendations. Approximately 24 h post-transfection, a portion of the cells were infected with NDV at an MOI of 5 for an additional 4 h, and western blotting was performed to detect the percentage NP protein. A portion of the cells were then fixed and immunofluorescent staining was performed using anti-EEA1 and anti-NP antibodies to assess co-localization the EEA1 and NP proteins.

### Statistical analysis

All experiments were performed independently three times. The data were analyzed using the student's *t-test*, and the results were presented as the mean ±standard deviation (SD). The significance levels were defined as *P* < 0.05.
